# Cytotoxicity and pro-apoptosis activity of synthetic 1,3-thiazole incorporated phthalimide derivatives on cancer cells

**DOI:** 10.22038/ijbms.2021.53845.12103

**Published:** 2021-05

**Authors:** Omid Tavallaei, Milad Heidarian, Marzieh Marzbany, Alireza Aliabadi

**Affiliations:** 1 Pharmaceutical Sciences Research Center, Health Institute, School of Pharmacy, Kermanshah University of Medical Sciences, Kermanshah, Iran; 2 Department of Pharmacognosy and Pharmaceutical Biotechnology, Faculty of Pharmacy, Kermanshah University of Medical Sciences, Kermanshah, Iran; 3 Students Research Committee, Faculty of Pharmacy, Kermanshah University of Medical Sciences, Kermanshah, Iran; 4 Department of Medicinal Chemistry, Faculty of Pharmacy, Kermanshah University of Medical Sciences, Kermanshah, Iran

**Keywords:** Apoptosis, Cancer cells, Cytotoxicity, Phthalimide, Thiazole

## Abstract

**Objective(s):**

Cancer is the second important reason for death worldwide. In spite of advances in cancer treatment, however, survival of patients stays weak. Therefore, there is a critical need for advancement of new anticancer drugs. Regarding the hopeful biological activity of phthalimide derivatives, in this study, synthesis, cytotoxicity, and pro-apoptosis activity of eleven derivatives of thiazole bearing phthalimide structure were evaluated.

**Materials and Methods:**

First, target derivatives were synthesized. All synthesized compounds were characterized by spectroscopic methods. Cytotoxicity and pro-apoptosis activity of the synthesized compounds were evaluated in MDA-MB-468, PC-12, and MCF-7 cancer cell lines by MTT assay, caspase-3 activity, and TUNEL assay. Finally, expression of BAX, BCL-2, and FAS (as markers of apoptosis) was assessed by the RT-PCR procedure.

**Results:**

Among the eleven compounds, **5b** (IC_50_ = 0.2±0.01 µM) was found to be the most potent derivative against MCF-7 cells. Also, Compound **5k** and **5g** showed strong cytotoxic activity against MDA-MB-468 and PC-12 cells with IC_50_ value of 0.6±0.04 µM and 0.43±0.06 µM, respectively. DNA fragmentation and activity of caspase-3 data suggest that cytotoxic activity of the compounds on cancer cells might be related to apoptosis. Also, RT-PCR of apoptosis markers indicated that these compounds induce apoptosis through the intrinsic pathway.

**Conclusion:**

Our findings suggest that *para* chloro derivative (**5c**) may be a promising agent for treatment of cancer cells by the targeted intrinsic pathway of apoptosis and could be used as a drug candidate for *in vivo* assessment in the treatment of cancer.

## Introduction

**Figure 1:**
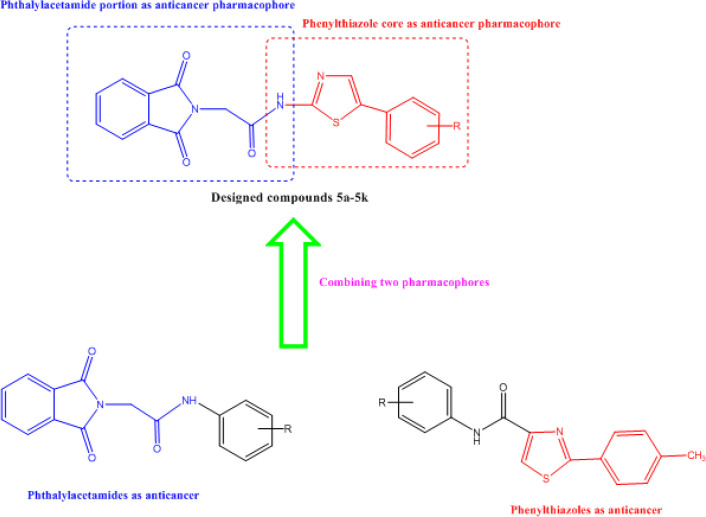
Design of target compounds **5a-5k.** Combining two pharmacophores namely phthalylacetamides and phenylthiazole led to the design of target compounds **5a-5k**

Cancer is a highly ordinary disorder (1). In the struggle versus cancer, exploration for new compounds with chemotherapeutic characteristics is widespread and numerous methods have been employed to obtain compounds comprising isolation from plants and animals and use of synthetic and combinatorial chemistry and molecular modeling (2-6). Phthalimides, a classical protecting group for amines with two carbonyl groups bound to a secondary amine or ammonia, have been widely utilized to synthesize molecules with many-sided pharmacological effects, including antimycobacterial, anticonvulsant, anticancer, anti-Alzheimer, anti-inflammatory, and analgesic (7-10). One of the most known phthalimide derivatives is thalidomide (Thl) and its analogues, multi-target compounds that can act as immunomodulators, angiogenesis inhibitors, and anticancer agent (for example, in human multiple myeloma, mammary neoplasia, liver cancer, oral squamous cell carcinoma, hormone-refractory prostate cancer) (6-21). Therefore, development of analogues and/or their administration in conjunction with other anticancer agents are under exploration in order to improve its efficacy and reduce toxicity. Besides, 1,3-thiazole as famous heterocyclic nucleus has exhibited potential cytotoxic activity via activation of caspases and subsequently induction of apoptosis. Therefore, numerous analogs based on 1,3-thiazole substructure and pharmacophore were developed in our previous works (22-25). The 1,3-thiazole heterocycle has appeared in some novel anticancer drugs such as dasatinib as tyrosine kinase inhibitor as well as ixabepilone as a microtubule stabilizer. Herein, due to incorporation of two promising anticancer pharmacophores that we reported previously, namely phthalimide and 1,3-thiazole, we hope to achieve a new series of anticancer derivatives (5a-5k) with remarkable potency (Figure 1).

The cytotoxic action of many chemotherapeutic drugs is due to their ability in apoptosis induction (26, 27). Apoptosis is the programmed cell death that happens during differentiation, development, in response to a variety of insults such as cytotoxic molecules or compounds, and in tumor cell deletion (28, 29). Cytotoxic drug-induced damage to the cells, particularly to the DNA, triggers apoptosis by either the intrinsic pathway or the extrinsic pathway (28). The extrinsic pathway is started at the plasma membrane by the interaction of cell-surface death receptors with their ligands (28, 30). Conversely, the intrinsic pathway, also known as the mitochondrial pathway, is triggered through the release of cytochrome c by mitochondria inside the cell (28, 31-35).

Therefore, in this study, we evaluated the anticancer potential of new phthalimide derivatives by the study of cytotoxicity and pro-apoptosis activity of these derivatives on human cancer cell lines. 

## Materials and Methods

***General procedure for synthesis of 5-phenylthiazol-2-amine derivatives (3a-3k)***

In order to synthesize and prepare **3a-3k** derivatives, first, in a 50 ml flat bottom balloon, 8.6 mmol of acetophenone derivatives (**1a-1k**), 8.6 mmol of iodine, 17.2 mmol of thiourea were added and mixed. Then, the reaction mixture was kept at 120 °C for 2 hr. After this time the reaction was completed (TLC reaction flow was tracked), and the reaction vessel was allowed to cool to 70 °C. Then, the reaction mixture was turned into powder, diethyl ether added to it, and the crude precipitate washed with ether. After the precipitate was dried, hot water was added to it and the pH of the resulting mixture was checked by adding NH_4_OH. The reaction mixture was passed through a strainer. The precipitate was dried and crystallized with a mixture of water and ethanol (1:4). Finally, the products (**3a-3k**) were obtained in pure form with high efficiency (Table 1) (36).

***5-(4-Fluorophenyl)thiazol-2-amine (3a)***

^1^HNMR (CDCl_3_, 250 MHz) δ: 5.24 (brs, NH_2_), 6.64 (s, 1H, H_4_-thiazole), 7.06 (m, 2H, H_2,6_-4-fluorophenyl), 7.72 (d, 2H, H_3,5_-fluorophenyl). ^13^CNMR (CDCl_3_, 62.9 MHz) δ: 102.1 (C_5_-thiazole), 115.5 (d, C_3,5_-4-fluorophenyl), 127.7 (d, C_2,6_-4-fluorophenyl), 130.9 (C_1_-phenyl), 150.1 (C_4_-thiazole), 162.4 (d, C_4_-4-fluorophenyl), 168.0 (C_2_-thiazole). IR (KBr, cm-1) ῡ: 3466, 3292 (stretch, NH), 3120 (stretch, C-H, aromatic), 2978, 2933 (stretch, C-H, aliphatic), 1635 (bend, NH). MS (m/z): 194 (M^+^).

***5-(2-Chlorophenyl)thiazol-2-amine (3b)***

^1^HNMR (CDCl_3_, 250 MHz) δ: 5.2 (brs, NH_2_), 6.72 (s, 1H, H_4_-thiazole), 7.28-7.38 (2H, 2-chlorophenyl), 7.77 (d, 2H, *J* = 7 Hz, H_3_-2-chlorophenyl). ^13^CNMR (CDCl_3_, 62.9 MHz) δ: 102.7 (C_5_-thiazole), 126.0 (C_5_-2-chlorophenyl), 127.8 (C_6_-2-chlorophenyl), 128.6 (C_3_-2-chlorophenyl), 134.5 (C_2_-2-chlorophenyl), 151.0 (C_1_-2-chlorophenyl), 152.2 (C_4_-thiazole), 162.4 (C_4_-4-chlorophenyl), 167.3 (C_2_-thiazole). IR (KBr, cm-1) ῡ: 3435, 3253 (stretch, NH), 3113, 3070 (stretch, C-H, aromatic), 2924 (stretch, C-H, aliphatic), 1598 (bend, NH). MS (m/z): 212 (M^+^+2), 210 (M^+^).

***5-(4-Chlorophenyl)thiazol-2-amine (3c)***

^1^HNMR (CDCl_3_, 250 MHz) δ: 5.10 (brs, NH_2_), 6.71 (s, 1H, H_4_-thiazole), 7.34 (d, 2H, *J* = 8 Hz, H_2,6_-4-chlorophenyl), 7.70 (d, 2H, *J* = 8 Hz, H_3,5_-4-chlorophenyl). ^13^CNMR (CDCl_3_, 62.9 MHz) δ: 103.2 (C_5_-thiazole), 127.3 (C_3,5_-4-chlorophenyl), 128.8 (C_2,6_-4-chlorophenyl), 133.1 (C_1_-phenyl), 150.1 (C_4_-thiazole), 162.4 (C_4_-4-chlorophenyl), 167.3 (C_2_-thiazole). IR (KBr, cm-1) ῡ: 3437, 3284 (stretch, NH), 3111 (stretch, C-H, aromatic), 2966, 2927 (stretch, C-H, aliphatic), 1633 (bend, NH). MS (m/z): 212 (M^+^+2), 210 (M^+^).

***5-(2-Methoxyphenyl)thiazol-2-amine (3d)***

^1^HNMR (CDCl_3_, 250 MHz) δ: 3.84 (s, 3H, -OCH_3_), 5.09 (brs, NH_2_), 6.72 (s, 1H, H_4_-thiazole), 6.84-7.15 (m, 2H, *J* = 7 Hz, H_3,5_-2-hydroxyphenyl), 7.50-7.73 (m, 2H, H_4,6_-2-hydroxyphenyl). ^13^CNMR (CDCl_3_, 62.9 MHz) δ: 55.9 (-OCH_3_), 116.2 (C_3_-2-methoxyphenyl), 129.1 (C_4_-2-methoxyphenyl), 128.9 (C_6_-2-methoxyphenyl), 121.2 (C_5_-2-methoxyphenyl), 121.5 (C_5_-thiazole), 122.7 (C_1_-2-methoxyphenyl), 140.1 (C_4_-thiazole), 156.9 (C_2_-2-methoxyphenyl), 167.7 (C_2_-thiazole). IR (KBr, cm^-1^) ῡ: 3436, 3302 (stretch, NH), 3115 (stretch, C-H, aromatic), 2995, 2939 (stretch, C-H, aliphatic), 1627 (bend, NH). MS (m/z): 206 (M^+^). 

***5-(3-Methoxyphenyl)thiazol-2-amine (3e)***

^1^HNMR (CDCl_3_, 250 MHz) δ: 3.85 (s, 3H, -OCH_3_), 5.43 (brs, NH_2_), 6.70 (s, 1H, H_4_-thiazole), 6.84 (d, 1H, *J* = 7 Hz, H_6_-3-methoxyphenyl), 7.28-7.33 (m, 3H, 3-methoxyphenyl). ^13^CNMR (CDCl_3_, 62.9 MHz) δ: 55.3 (-OCH_3_), 103.0 (C_2_-3-methoxyphenyl), 111.4 (C_4_-3-methoxyphenyl), 113.7 (C_6_-3-methoxyphenyl), 118.5 (C_5_-thiazole), 129.6 (C_5_-3-methoxyphenyl), 136.0 (C_1_-4-methoxyphenyl), 151.0 (C_4_-thiazole), 159.8 (C_3_-4-methoxyphenyl), 167.6 (C_2_-thiazole). IR (KBr, cm^-1^) ῡ: 3400, 3302 (stretch, NH), 3120 (stretch, C-H, aromatic), 2997, 2941 (stretch, C-H, aliphatic), 1643 (bend, NH). MS (m/z): 206 (M^+^).

***5-(4-Methoxyphenyl)thiazol-2-amine (3f)***

^1^HNMR (DMSO-d_6_, 250 MHz) δ: 3.83 (s, 3H, -OCH_3_), 5.10 (brs, NH_2_), 6.58 (s, 1H, H_4_-thiazole), 6.91 (d, 2H, *J* = 8 Hz, H_3,5_-4-methoxyphenyl), 7.71 (d, 2H, *J* = 8 Hz, H_2,6_-4-methoxyphenyl). ^13^CNMR (CDCl_3_, 62.9 MHz) δ: 55.3 (-OCH_3_), 101.03 (C_3,5_-4-methoxyphenyl), 114.0 (C_5_-thiazole), 127.3 (C_2,6_-4-methoxyphenyl), 133.1 (C_1_-4-methoxyphenyl), 150.1 (C_4_-thiazole), 158.4 (C_4_-4-methoxyphenyl), 167.3 (C_2_-thiazole). IR (KBr, cm^-1^) ῡ: 3437, 3267 (stretch, NH), 3115 (stretch, C-H, aromatic), 2995, 2962, 2931 (stretch, C-H, aliphatic), 1625 (bend, NH). MS (m/z): 206 (M^+^). 

***2-(2-Aminothiazol-5-yl)phenol (3g)***

^1^HNMR (CDCl_3_, 250 MHz) δ: 5.34 (brs, NH_2_), 6.72 (s, 1H, H_4_-thiazole), 7.01-7.12 (m, 1H, *J* = 7.5 Hz, H_3,5_-2-hydroxyphenyl), 7.31-7.60 (m, 2H, H_4,6_-3-hydroxyphenyl), 8.59 (brs, OH). ^13^CNMR (CDCl_3_, 62.9 MHz) δ: 118.3 (C_3_-2-hydroxyphenyl), 121.9 (C_5_-2-hydroxyphenyl), 122.9 (C_5_-thiazole), 124.4 (C_1_-2-hydroxyphenyl), 129.4 (C_6_-2-hydroxyphenyl), 131.2 (C_4_-2-hydroxyphenyl), 137.3 (C_4_-thiazole), 155.7 (C_2_-2-hydroxyphenyl), 168.6 (C_2_-thiazole). IR (KBr, cm^-1^) ῡ: 3327(stretch, NH), 2927, 2854 (stretch, C-H, aliphatic), 2500-3500 (stretch, OH, broad), 1627 (bend, NH). MS (m/z): 192 (M^+^).

***3-(2-Aminothiazol-5-yl)phenol (3h)***

^1^HNMR (CDCl_3_, 250 MHz) δ: 5.35 (brs, NH_2_), 6.70 (s, 1H, H_4_-thiazole), 6.81 (d, 1H, *J* = 7 Hz, H_6_-3-hydroxyphenyl), 7.21-7.35 (m, 3H, 3-hydroxyphenyl), 8.59 (brs, OH). ^13^CNMR (CDCl_3_, 62.9 MHz) δ: 104.1 (C_2_-3-hydroxyphenyl), 113.7 (C_4_-3-hydroxyphenyl), 114.1 (C_6_-3-hydroxyphenyl), 119.5 (C_5_-thiazole), 129.9 (C_5_-3-hydroxyphenyl), 136.3 (C_1_-4-hydroxyphenyl), 151.3 (C_4_-thiazole), 157.9 (C_3_-4-hydroxyphenyl), 168.6 (C_2_-thiazole). IR (KBr, cm^-1^) ῡ: 3361(stretch, NH), 2956, 2924, 2854 (stretch, C-H, aliphatic), 2500-3600 (stretch, OH, broad), 1604 (bend, NH). MS (m/z): 192 (M^+^).

***4-(2-Aminothiazol-5-yl)phenol (3i)***

^1^HNMR (DMSO-d_6_, 250 MHz) δ: 5.1 (brs, NH_2_), 6.71 (d, 2H, *J* = 8 Hz, H_3,5_-4-hydoxyphenyl), 6.84 (s, 1H, H_4_-thiazole), 7.37 (d, 2H, *J* = 8 Hz, H_2,6_-4-hydroxyphenyl), 8.62 (brs, OH). ^13^CNMR (CDCl_3_, 62.9 MHz) δ: 102.12 (C_3,5_-4-hydroxyphenyl), 114.4 (C_5_-thiazole), 125.4 (C_1_-4-hydroxyphenyl), 128.5 (C_2,6_-4-hydroxyphenyl), 150.7 (C_4_-thiazole), 157.3 (C_4_-4-hydroxyphenyl), 168.2 (C_2_-thiazole). IR (KBr, cm^-1^) ῡ: 3363, 3209 (stretch, NH), 3115 (stretch, C-H, aromatic), 2927, 2819 (stretch, C-H, aliphatic), 1610 (bend, NH). MS (*m/z*): 192 (M^+^).

***5-(4-Nitrophenyl)thiazol-2-amine (3j)***

^1^HNMR (CDCl_3_, 250 MHz) δ: 5.26 (brs, NH_2_), 6.66 (s, 1H, H_4_-thiazole), 7.98 (m, 2H, H_2,6_-4-nitrophenyl), 8.21 (d, 2H, H_3,5_-nitrophenyl). ^13^CNMR (DMSO-d_6_, 62.9 MHz) δ: 122.9 (C_5_-thiazole), 124.6 (C_3,5_-4-nitrophenyl), 128.8 (C_2,6_-4-nitrophenyl), 138.1 (C_4_-thiazole), 139.5 (C_1_-4-nitrophenyl), 148.6 (C_4_-4-nitrophenyl), 169.2 (C_2_-thiazole). IR (KBr, cm^-1^) ῡ: 3327, 3292 (stretch, NH), 3103 (stretch, C-H, aromatic), 2927, 2854 (stretch, C-H, aliphatic), 1627 (bend, NH), 1516, 1344 (stretch, NO_2_). MS (m/z, %): 221 (M^+^).

***5-(4-Bromophenyl)thiazol-2-amine (3k)***

^1^HNMR (CDCl_3_, 250 MHz) δ: 5.06 (brs, NH_2_), 6.72 (s, 1H, H_4_-thiazole), 7.49 (d, 2H, J = 8.5 Hz, H_2,6_-4-bromophenyl), 7.65 (d, 2H, J = 8.5 Hz, H_3,5_-4-bromophenyl). ^13^CNMR (DMSO-d_6_, 62.9 MHz) δ: 102.9 (C_5_-thiazole), 120.6 (C_3,5_-4-bromophenyl), 128.0 (C_2,6_-4-bromophenyl), 131.8 (C_4_-thiazole), 134.5 (C_1_-4-bromophenyl), 149.0 (C_4_-4-bromophenyl), 168.8 (C_2_-thiazole). IR (KBr, cm^-1^) ῡ: 3427, 3282 (stretch, NH), 3111 (stretch, C-H, aromatic), 2966, 2929 (stretch, C-H, aliphatic), 1633 (bend, NH), 1516, 1344 (stretch, NO_2_). MS (m/z, %): 255 (M^+^+2), 253 (M^+^).

***General procedure for synthesis of 2-(1,3-Dioxoisoindolin-2-yl)-N-(5-phenylthiazol-2-yl)acetamide derivatives (5a-5k)***

In order to synthesize compounds **5a-5k**, appropriate derivatives of (**3a-3k**), 2-(1,3-dioxoisoindolin-2-yl)acetic acid (4), DCC, and HOBt were mixed equivalently in 20 ml of THF solvent in a 50 ml flask, and the resulting mixture put in an ice bath for 1 hr. Then, the resulting mixture was kept at room temperature for 24 hr. The reaction process was controlled by TLC. After reaction completion and product formation, the reaction mixture smooth. The resulting solution was dried by rotary apparatus. Then, water and ethyl acetate were added to it and the organic phase separated, and washed two times with aqueous sodium chloride solution (brine). The separated organic phase was dewatered by addition of anhydrous sodium sulfate and then filtered. The resulting solution was dried by removing the solvent using a rotary apparatus; any impurity was removed by washing with diethyl ether. Finally, the product was obtained with high purity and efficiency (Table 1) (37, 38).

***2-(1,3-Dioxoisoindolin-2-yl)-N-(5-(4-fluorophenyl)thiazol-2-yl)acetamide (5a)***

^1^HNMR (CDCl_3_, 250 MHz) δ: 4.36 (s, 2H, -CH_2_-), 7.36-7.52 (m, 4H, 4-fluorophenyl), 7.35 (s, 1H, H_4_-thiazole), 7.72 (m, 2H, H_5,6_-phthalimide), 7.85 (m, 2H, H_4,7_-phthalimide). ^13^CNMR (DMSO-d_6_, 62.9 MHz) δ: 47.9 (-CH_2_), 122.6 (C_5_-thiazole), 117.3 (C_3,5_-4-fluorophenyl), 124.4 (C_4,7_-phthalimide), 129.5 (C_1_-4-fluorophenyl), 129.8 (C_2,6_-4-fluorophenyl), 132.1 (C_4a,7a_-phthalimide), 132.6 (C_5,6_-phthalimide), 137.5 (C_4_-thiazole), 162.9 (C_2_-thiazole), 163.7 (C_4_-4-fluorophenyl), 168.7 (C=O, phthalimide), 169.3 (C=O, amide). IR (KBr, cm^-1^) ῡ: 3325 (stretch, NH), 3064 (stretch, C-H, aromatic), 2927, 2850 (stretch, C-H, aliphatic), 1722 (stretch, C=O, phthalimide), 1627 (stretch, C=O, amide). MS (m/z, %): 381 (M^+^, 5), 264 (30), 233 (40), 221 (55), 194 (100), 160 (60), 152 (80), 133 (20), 107 (35), 71 (30), 43 (30).

***N-(5-(2-Chlorophenyl)thiazol-2-yl)-2-(1,3-dioxoisoindolin-2-yl)acetamide (5b)***

^1^HNMR (CDCl_3_, 250 MHz) δ: 4.35 (s, 2H, -CH_2_-), 7.08-7.32 (m, 4H, 2-chlorophenyl), 7.42 (s, 1H, H_4_-thiazole), 7.71 (m, 2H, H_5,6_-phthalimide), 7.82 (m, 2H, H_4,7_-phthalimide), 8.73 (brs, NH). ^13^CNMR (DMSO-d_6_, 62.9 MHz) δ: 48.0 (-CH_2_), 110.1 (C_5_-thiazole), 119.3 (C_5_-2-chlorophenyl), 123.8 (C_4,7_-phthalimide), 124.9 (C_6_-2-chlorophenyl), 128.7 (C_4a,7a_-phthalimide), 128.3 (C_3_-2-chlorophenyl), 129.0 (C_5,6_-phthalimide), 131.9 (C_4_-thiazole), 134.5 (C_2_-2-chlorophenyl), 135.3 (C_1_-2-chlorophenyl), 136.8 (C_4_-2-chlorophenyl), 162.9 (C_2_-thiazole), 168.7 (C=O, phthalimide), 169.3 (C=O, amide). IR (KBr, cm^-1^) ῡ: 3325 (stretch, NH), 3059 (stretch, C-H, aromatic), 2927, 2873 (stretch, C-H, aliphatic), 1714 (stretch, C=O, phthalimide), 1625 (stretch, C=O, amide). MS (m/z, %): 399 (M^+^+2, 4), 397 (M^+^, 10), 313 (15), 280 (40), 251 (55), 237 (60), 210 (80), 167 (20), 160 (45), 133 (45), 71 (100), 43 (65). 

***N-(5-(4-Chlorophenyl)thiazol-2-yl)-2-(1,3-dioxoisoindolin-2-yl)acetamide (5c)***

^1^HNMR (CDCl_3_, 250 MHz) δ: 4.36 (s, 2H, -CH_2_-), 7.35-7.42 (m, 4H, 4-chlorophenyl), 7.46 (s, 1H, H_4_-thiazole), 7.74 (m, 2H, H_5,6_-phthalimide), 7.85 (m, 2H, H_4,7_-phthalimide). ^13^CNMR (DMSO-d_6_, 62.9 MHz) δ: 47.9 (-CH_2_), 110.1 (C_5_-thiazole), 119.3 (C_3,5_-4-chlorophenyl), 123.7 (C_4,7_-phthalimide), 125.0 (C_2,6_-4-chlorophenyl), 127.8 (C_4a,7a_-phthalimide), 128.8 (C_5,6_-phthalimide), 130.6 (C_4_-thiazole), 132.5 (C_1_-4-chlorophenyl), 135.3 (C_4_-4-chlorophenyl), 162.9 (C_2_-thiazole), 168.7 (C=O, phthalimide), 169.3 (C=O, amide). IR (KBr, cm^-1^) ῡ: 3325 (stretch, NH), 3088 (stretch, C-H, aromatic), 2929, 2852 (stretch, C-H, aliphatic), 1712 (stretch, C=O, phthalimide), 1629 (stretch, C=O, amide). MS (m/z, %): 399 (M^+^+2, 2), 397 (M^+^, 5), 313 (10), 280 (60), 251 (65), 237 (70), 210 (90), 167 (40), 160 (55), 133 (25), 71 (100), 43 (85). 

***2-(1,3-Dioxoisoindolin-2-yl)-N-(5-(2-methoxyphenyl)thiazol-2-yl)acetamide (5d)***

^1^HNMR (CDCl_3_, 250 MHz) δ: 3.77 (s, 3H, -OCH_3_), 4.39 (s, 2H, -CH_2_-), 7.45 (s, 1H, H_4_-thiazole), 7.09-7.27 (m, 2H, H_3,5_-2-methoxyphenyl), 7.51-7.69 (m, 2H, H_4,6_-2-methoxyphenyl), 7.72 (m, 2H, H_5,6_-phthalimide), 7.86 (m, 2H, H_4,7_-phthalimide). ^13^CNMR (DMSO-d_6_, 62.9 MHz) δ: 47.9 (-CH_2_-), 104.2 (C_5_-thiazole), 115.7 (C_4,7_-phthalimide), 118.8 (C_3_-2-methoxyphenyl), 121.1 (C_5_-2-methoxyphenyl), 123.9 (C_1_-2-methoxyphenyl), 124.2 (C_5_-thiazole), 125.2 (C_4a,7a_-phthalimide), 126.9 (C_5,6_-phthalimide), 127.9 (C_6_-2-methoxyphenyl), 130.8 (C_4_-2-methoxyphenyl), 131.3 (C_4_-thiazole), 158.2 (C_2_-2-methoxyphenyl), 157.8 (C_2_-thiazole), 167.6 (C=O, amide), 168.5 (C=O, phthalimide). IR (KBr, cm^-1^) ῡ: 3323 (stretch, NH), 3038 (stretch, C-H, aromatic), 2927, 2850 (stretch, C-H, aliphatic), 1720 (stretch, C=O, phthalimide), 1628 (stretch, C=O, amide). MS (m/z, %): 393 (M^+^, 12), 276 (55), 245 (35), 206 (100), 194 (15), 149 (30), 121 (35), 71 (25), 43 (30). 

***2-(1,3-Dioxoisoindolin-2-yl)-N-(5-(3-methoxyphenyl)thiazol-2-yl)acetamide (5e)***

^1^HNMR (CDCl_3_, 250 MHz) δ: 3.78 (s, 3H, -OCH_3_), 4.42 (s, 2H, -CH_2_-), 7.45 (s, 1H, H_4_-thiazole), 7.04-7.27 (m, 2H, H_2,4_-3-methoxyphenyl), 7.42-7.50 (m, 2H, H_5,6_-3-methoxyphenyl), 7.70 (m, 2H, H_5,6_-phthalimide), 7.87 (m, 2H, H_4,7_-phthalimide). IR (KBr, cm^-1^) ῡ: 3325 (stretch, NH), 3034 (stretch, C-H, aromatic), 2927, 2850 (stretch, C-H, aliphatic), 1720 (stretch, C=O, phthalimide), 1625 (stretch, C=O, amide). ^13^CNMR (DMSO-d_6_, 62.9 MHz) δ: 47.5 (-CH_2_-), 55.3 (-OCH_3_), 11.7 (C_2_-3-methoxyphenyl), 115.2 (C_4_-3-methoxyphenyl), 117.8 (C_6_-3-methoxyphenyl), 122.7 (C_5_-thiazole), 124.2 (C_4,7_-phthalimide), 130.2 (C_5_-3-methoxyphenyl), 131.8 (C_4a,7a_-phthalimide), 132.6 (C_5,6_-phthalimide), 134.9 (C_1_-3-methoxyphenyl), 137.3 (C_4_-thiazole), 159.8 (C_3_-3-methoxyphenyl), 163.2 (C_2_-thiazole), 169.3 (C=O, phthalimide), 169.8 (C=O, amide). MS (m/z, %): 393 (M^+^, 5), 276 (20), 245 (30), 206 (100), 194 (45), 149 (25), 121 (35), 71 (40), 43 (25). 

***2-(1,3-Dioxoisoindolin-2-yl)-N-(5-(4-methoxyphenyl)thiazol-2-yl)acetamide (5f)***

^1^HNMR (CDCl_3_, 250 MHz) δ: 3.75 (s, 3H, -OCH_3_), 4.42 (s, 2H, -CH_2_-), 6.67 (d, 2H, H_3,5_-4-methoxyphenyl), 6.90 (d, 2H, H_3,5_-4-methoxyphenyl), 7.42 (s, 1H, H_4_-thiazole), 7.67 (m, 2H, H_5,6_-phthalimide), 7.89 (m, 2H, H_4,7_-phthalimide). ^13^CNMR (DMSO-d_6_, 62.9 MHz) δ: 48.1 (-CH_2_-), 103.9 (C_5_-thiazole), 110.8 (C_2,6_-4-methoxyphenyl), 115.4 (C_4,7_-phthalimide), 118.9 (C_1_-4-methoxyphenyl), 124.2 (C_3,5_-4-methoxyphenyl), 125.0 (C_4a,7a_-phthalimide), 127.4 (C_5,6_-phthalimide), 130.9 (C_4_-thiazole), 157.8 (C_4_-4-methoxyphenyl), 158.1 (C_2_-thiazole), 168.7 (C=O, phthalimide), 169.7 (C=O, amide). IR (KBr, cm^-1^) ῡ: 3325 (stretch, NH), 3088 (stretch, C-H, aromatic), 2929, 2852 (stretch, C-H, aliphatic), 1720 (stretch, C=O, phthalimide), 1625 (stretch, C=O, amide). MS (m/z, %): 393 (M^+^, weak), 276 (45), 245 (45), 206 (100), 194 (35), 149 (45), 121 (25), 71 (20), 43 (15). 

***2-(1,3-Dioxoisoindolin-2-yl)-N-(5-(2-hydroxyphenyl)thiazol-2-yl)acetamide (5g)***

^1^HNMR (DMSO-d_6_, 250 MHz) δ: 4.26 (s, 2H, -CH_2_-), 6.85-7.18 (m, 4H, 2-hydroxyphenyl), 7.35 (s, 1H, H_4_-thiazole), 7.42 (m, 2H, H_5,6_-phthalimide), 7.63 (m, 2H, H_4,7_-phthalimide), 7.89 (brs, OH). ^13^CNMR (DMSO-d_6_, 62.9 MHz) δ: 48.0 (-CH_2_-), 103.6 (C_5_-thiazole), 115.9 (C_4,7_-phthalimide), 117.8 (C_3_-2-hydroxyphenyl), 121.6 (C_5_-2-hydroxyphenyl), 123.3 (C_1_-2-hydroxyphenyl), 123.8 (C_5_-thiazole), 124.8 (C_4a,7a_-phthalimide), 127.4 (C_5,6_-phthalimide), 128.5 (C_6_-2-hydroxyphenyl), 130.1 (C_4_-2-hydroxyphenyl), 130.8 (C_4_-thiazole), 157.2 (C_2_-2-hydroxyphenyl), 157.8 (C_2_-thiazole), 167.8 (C=O, amide), 168.7 (C=O, phthalimide). IR (KBr, cm^-1^) ῡ: 3329 (stretch, NH), 3041 (stretch, C-H, aromatic), 2927, 2875 (stretch, C-H, aliphatic), 1720 (stretch, C=O, phthalimide), 1627 (stretch, C=O, amide). MS (m/z, %): 379 (M^+^, 10), 332 (20), 299 (25), 262 (35), 233 (45), 219 (60), 192 (100), 188 (45), 121 (35), 71 (30), 43 (25). 

***2-(1,3-Dioxoisoindolin-2-yl)-N-(5-(3-hydroxyphenyl)thiazol-2-yl)acetamide (5h)***

^1^HNMR (DMSO-d_6_, 250 MHz) δ: 4.24 (s, 2H, -CH_2_-), 6.73-6.97 (m, 4H, 3-hydroxyphenyl), 7.34 (s, 1H, H_4_-thiazole), 7.42 (m, 2H, H_5,6_-phthalimide), 7.63 (m, 2H, H_4,7_-phthalimide), 7.87 (brs, OH), 9.50 (brs, NH). ^13^CNMR (DMSO-d_6_, 62.9 MHz) δ: 48.0 (-CH_2_-), 110.4 (C_4,7_-phthalimide), 112.6 (C_2_-3-hydroxyphenyl), 115.3 (C_4_-3-hydroxyphenyl), 119.3 (C_6_-3-hydroxyphenyl), 123.8 (C_5_-thiazole), 124.4 (C_4a,7a_-phthalimide), 126.7 (C_5,6_-phthalimide), 130.1 (C_5_-3-hydroxyphenyl), 131.1 (C_4_-thiazole), 131.9 (C_1_-3-hydroxyphenyl), 157.8 (C_3_-3-hydroxyphenyl), 157.2 (C_2_-thiazole), 167.8 (C=O, amide), 169.5 (C=O, phthalimide). IR (KBr, cm^-1^) ῡ: 3336 (stretch, NH), 3064 (stretch, C-H, aromatic), 2927, 2852 (stretch, C-H, aliphatic), 1718 (stretch, C=O, phthalimide), 1625 (stretch, C=O, amide). MS (m/z, %): 379 (M^+^, 15), 332 (20), 299 (10), 262 (35), 233 (50), 219 (70), 192 (100), 188 (25), 121 (35), 71 (45), 43 (35). 

***2-(1,3-Dioxoisoindolin-2-yl)-N-(5-(4-hydroxyphenyl)thiazol-2-yl)acetamide (5i)***

^1^HNMR (DMSO-d_6_, 250 MHz) δ: 4.28 (s, 2H, -CH_2_-), 6.77 (d, 2H, H_3,5_-4-hydroxyphenyl), 7.36 (s, 1H, H_4_-thiazole), 7.48 (m, 2H, H_5,6_-phthalimide), 7.52 (d, 2H, H_2,6_-4-hydroxyphenyl), 7.68 (m, 2H, H_4,7_-phthalimide), 7.89 (brs, OH), 9.62 (brs, NH). ^13^CNMR (DMSO-d_6_, 62.9 MHz) δ: 48.0 (-CH_2_-), 103.5 (C_5_-thiazole), 110.2 (C_2,6_-4-hydroxyphenyl), 115.4 (C_4,7_-phthalimide), 119.5 (C_1_-4-hydroxyphenyl), 123.8 (C_3,5_-4-hydroxyphenyl), 124.8 (C_4a,7a_-phthalimide), 127.4 (C_5,6_-phthalimide), 130.3 (C_4_-thiazole), 157.2 (C_4_-4-hydroxyphenyl), 157.8 (C_2_-thiazole), 168.7 (C=O, phthalimide), 169.4 (C=O, amide). IR (KBr, cm^-1^) ῡ: 3336 (stretch, NH), 3064 (stretch, C-H, aromatic), 2927, 2852 (stretch, C-H, aliphatic), 1714 (stretch, C=O, phthalimide), 1625 (stretch, C=O, amide). MS (m/z, %): 379 (M^+^, 10), 332 (10), 299 (15), 262 (55), 233 (60), 219 (80), 192 (100), 188 (45), 121 (25), 71 (40), 43 (35). 

***2-(1,3-Dioxoisoindolin-2-yl)-N-(5-(4-nitrophenyl)thiazol-2-yl)acetamide (5j)***

^1^HNMR (DMSO-d_6_, 250 MHz) δ: 4.38 (s, 2H, -CH_2_-), 7.89 (d, 2H, H_2,6_-4-nitrophenyl), 8.22 (d, 2H, H_3,5_-4-nitrophenyl), 7.38 (s, 1H, H_4_-thiazole), 7.74 (m, 2H, H_5,6_-phthalimide), 7.88 (m, 2H, H_4,7_-phthalimide). ^13^CNMR (DMSO-d_6_, 62.9 MHz) δ: 49.4 (-CH_2_-), 103.9 (C_5_-thiazole), 110.9 (C_2,6_-4-nitrophenyl), 123.5 (C_4,7_-phthalimide), 123.9 (C_3,5_-4-nitrophenyl), 126.7 (C_3,5_-4-bromophenyl), 129.6 (C_4a,7a_-phthalimide), 132.05 (C_5,6_-phthalimide), 134.2 (C_1_-4-nitrophenyl), 148.0 (C_4_-4-nitrophenyl), 149.0 (C_4_-thiazole), 167.8 (C_2_-thiazole), 168.7 (C=O, phthalimide), 169.4 (C=O, amide). IR (KBr, cm^-1^) ῡ: 3325 (stretch, NH), 3032 (stretch, C-H, aromatic), 2927, 2850 (stretch, C-H, aliphatic), 1710 (stretch, C=O, phthalimide), 1628 (stretch, C=O, amide). MS (m/z, %): 408 (M^+^, 15), 248 (35), 220 (45), 188 (20), 179 (30), 160 (60), 71 (100), 43 (65). 

***N-(5-(4-Bromophenyl)thiazol-2-yl)-2-(1,3-dioxoisoindolin-2-yl)acetamide (5k)***

^1^HNMR (CDCl_3_, 250 MHz) δ: 4.35 (s, 2H, -CH_2_-), 7.37-7.47 (m, 4H, 4-bromophenyl), 7.42 (s, 1H, H_4_-thiazole), 7.73 (m, 2H, H_5,6_-phthalimide), 7.86 (m, 2H, H_4,7_-phthalimide). ^13^CNMR (DMSO-d_6_, 62.9 MHz) δ: 48.0 (-CH_2_-), 103.5 (C_5_-thiazole), 109.07 (C_2,6_-4-bromophenyl), 110.2 (C_4,7_-phthalimide), 119.5 (C_1_-4-bromophenyl), 123.8 (C_3,5_-4-bromophenyl), 124.7 (C_4a,7a_-phthalimide), 127.4 (C_5,6_-phthalimide), 130.9 (C_4_-thiazole), 162.5 (C_4_-4-bromophenyl), 167.7 (C_2_-thiazole), 168.7 (C=O, phthalimide), 169.4 (C=O, amide). IR (KBr, cm^-1^) ῡ: 3325 (stretch, NH), 3032 (stretch, C-H, aromatic), 2927, 2850 (stretch, C-H, aliphatic), 1714 (stretch, C=O, phthalimide), 1625 (stretch, C=O, amide). MS (m/z, %): 442 (M^+^+2, weak), 440 (M^+^, weak), 368 (10), 326 (30), 295 (38), 293 (40), 254 (30), 214 (35), 172 (55), 160 (50), 71 (100), 43 (75). 

***Cell culture***

 Human cancer MCF7, MDA-MB468, and PC12 cells were cultured in DMEM/F12 medium (Gibco, USA) supplemented with 10% fetal bovine serum (Sigma-Aldrich, USA) and 1% penicillin/streptomycin (Sigma-Aldrich, USA). Cell growth was carried out in a humidified atmosphere containing 5% CO_2_ and 95% air at 37 °C. When the confluency of cells reached more than 70%, they were washed with PBS and incubated with the trypsin-EDTA solution for 3 min at 37 °C to detach them from the flask. The cells were then re-suspended in the culture medium for seeding. The medium was changed 2–3 days and was sub-cultured when the cell population density reached 70–80% confluence.

***Cell viability assay***

Inhibition of cell proliferation was measured by colorimetric MTT assay. Briefly, cells were seeded into 96-well plates at a density of 20000 cells/well. The following day, cells were treated with different concentrations of compounds (1 μM, 0.5 μM, 0.25 μM, and 0.125 μM) and analyzed for cell viability after 24 hr. 5 mg/ml thiazolyl blue tetrazolium bromide (Sigma, Germany) was added to each well at a final concentration of 0.5 mg/ml and the plates were incubated at 37 °C for 2 hr. Medium containing unconverted MTT was removed and 100 µl MTT was added to each well. The plate was gently rotated in the dark for 15 min, before the measurements of absorbance at 570 nm by an Infinite F500 (Tecan, Austria) plate reader device. 

***TUNEL assay***

The DNA fragmentation in three cancer cell lines was detected using the colorimetric apoptosis detection kit (Titer TACS, R&D System, USA) following the manufacturer’s instructions. The cells were incubated overnight in 96-well culture plates, replaced with fresh media, and then treated with different IC_50_ concentrations of **5c** compound for 24 hr. Briefly, the cells were fixed with 3.7% buffered formaldehyde solution for 7 min, washed with PBS, permeabilized with 100% methanol for 20 min, washed twice with PBS, digested with proteinase K for 15 min, quenched with 3% hydrogen peroxide, washed with distilled water, labeled with the TdT reaction mix and incubated at 37 °C for 1 hr in a humidified chamber and the reaction terminated with stop buffer. Then the cells were incubated with Strep-HRP for 10 min, washed four times with PBS, followed by addition of TACS-Sapphire™ substrate, and the colorimetric reaction was stopped with 0.2 N HCl after 30 min. Negative controls were labeled without the TdT enzyme and positive controls were generated with TACS-Nuclease™ to create DNA breaks. The colorimetric reaction was measured in a spectrophotometer (BioTek Synergy, USA) at 450 nm and 630 nm absorbance.

***Caspase-3 activity***

For analysis of caspase activity, 4×10^4^ cells were plated overnight on a 96-well luminometer plate and allowed to attach overnight. The cells were treated with different IC_50_ concentrations of **5c** compound and incubated for a period of 24 hr. Then, the caspase-3 assay was performed according to the protocol of the Caspase-3 Colorimetric Assay Kit (Promega, USA) manufacturer. The assay was conducted in triplicates and caspase-3 activity was reported in 400–450 nm by the spectrophotometer (BioTek Synergy, USA).

***RNA extraction and cDNA synthesis***

Total RNA was extracted from 1 x 10^7^ cells using SinaPure™ RNA (Sinaclon, Iran), according to the manufacturer’s instructions. The resulting RNA pellet was dissolved in a maximum of 50 μl RNase free water and stored in aliquots at -80 °C until use. The integrity of the RNA was confirmed in randomly selected samples via agarose gel electrophoresis. Following RNA extraction, the total quantity of RNA from cells was utilized for first-strand cDNA synthesis. cDNA synthesis was performed using the First Strand cDNA synthesis Kit (SinaClon, Iran). The reaction mix contained 1 µg RNA, 1 µl oligo(dT), 15–20 pmol reverse transcription primer, 1 µl dNTP mix, 0.5 µl M-MuLV reverse transcriptase RNase H, 2 µl 10X buffer M-MuLV, 0.5 µl RNase inhibitor and diethylpyrocarbonate-treated (DEPC) water to a total volume of 10 µl. The reverse transcription reaction was incubated at 42 °C for 60 min and was terminated by incubation at 85 °C for 5 min.

***Quantitative (q) polymerase chain reaction (PCR)***

Gene-specific primers were designed and synthesized by Pishgam biotech co. Iran for amplification of the mRNA transcripts of Bax, Bcl-2, and FasL genes, as well as for Glyceraldehyde 3-phosphate dehydrogenase (GADPH(, which was used as a reference gene. The Bcl-2 specific primers were: forward 5′-TTGTGGCCTTCTTTGAGTTCGGTG-3′ and reverse 5′-GGTGCCGGTTCAGGTACTCAGTCA-3′, producing a 114 base pair (bp) PCR amplicon, the Bax specific primers were: forward 5′- CCTGTGCACCAAGGTGCCGGAACT-3′ and reverse 5′-CCACCCTGGTCTTGGATCCAGCCC-3′, generating an amplicon of 99 bp and FasL primers were: forward 5′-GCAATGAAACTGGGCTGTACTTT-3′ and reverse 5′- GGAGTTCCTCATGTAGACCTTGT -3′, resulting in a product of 101 bp.

Reverse transcription-qPCR analysis was performed using a thermal cycler (Corbet Research, UK). The reaction mixture contained 1 µg cDNA, 1 µl primers, 10 µl qPCR Master Mix and DEPC water in a total volume of 20 µl. The reactions were performed in duplicate under the following conditions: 95 °C for 15 min as a polymerase activation step, 40 cycles of 95 °C for 45 sec for denaturation, and 60 °C for 1 min for primer annealing, extension, and fluorescence detection. 

Gene expression analysis was performed using the comparative Ct (2−ΔΔCt) method. Briefly, the PCR products were detected by measuring the emitted fluorescence (Rn) at the end of each reaction cycle and average CT values were calculated for subsequent expression analysis. The threshold cycle (Ct) corresponds to the number of cycles required to detect a fluorescence signal above the baseline. The relative quantification units (RQ units = 2−ΔΔCt), representative of the normalized expression of the target genes, were calculated for each sample. ΔΔCt is the difference between the ΔCt value of a cartilage tissue sample and the ΔCT for the calibrator sample, whereas ΔCt is the difference between the Ct value of the target gene (Bcl-2, FasL, or Bax) and the Ct of the endogenous reference gene GADPH.

***Statistical analysis***

Experiments were performed at least in biological triplicates and graphs represent mean±SEM values. All statistical analyses were performed using GraphPad PRISM 8.0 software (GraphPad Software, Germany), using two-tailed t-tests or one-sample t-tests, as appropriate. *P*-values of *P*<0.05 were considered to be statistically significant (*), *P*<0.01 (**), *P*<0.001 (***), and *P*<0.0001 (****).

## Results

***Cytotoxicity effects of synthetic phthalimide derivatives on cancer cell lines***

**Figure 2:**
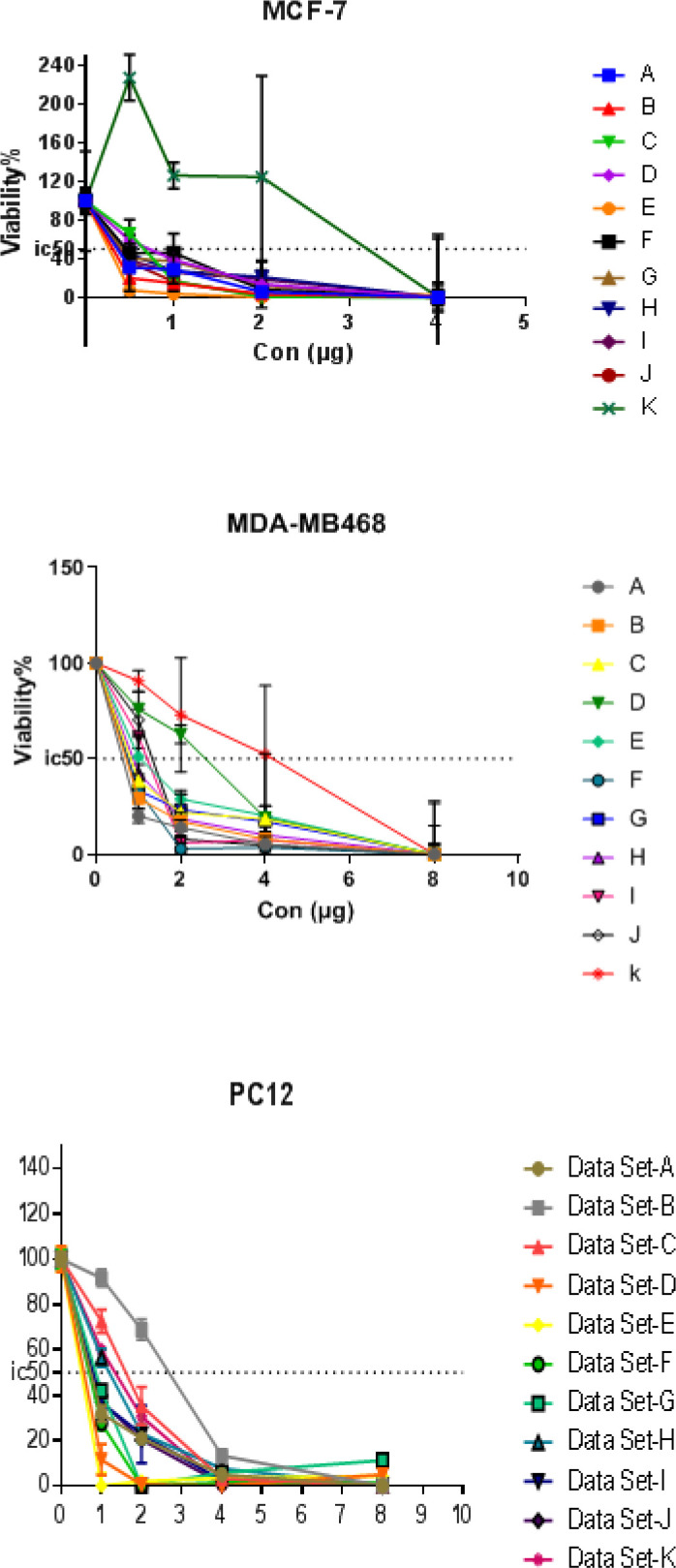
The viability A) MCF-7, B) MDA-MB468, and C) PC12 cell lines treated with the different concentrations of synthetic phthalimide derivatives for 24 hr after treatment in contrast with the control group. Results are expressed as Mean±SEM of three independent experiments (n=3)

The cytotoxicity effects of synthetic phthalimide derivatives on MCF-7, MDA-MB-468, and PC-12 cell lines were evaluated through MTT assay. Multiple concentrations of phthalimide derivatives were used and effective doses were calculated from the dose-response curve. Results of cytotoxicity evaluation against MCF-7, MDA-MB-468, and PC-12 cell lines of phthalimide derivatives are shown in Figure 2 and ​Table 2. Among the eleven investigated compounds, 5b (IC_50_ = 0.2±0.01 µM) was found to be the most potent derivative against MCF-7 cells. Also, Compounds 5k and 5g showed strong cytotoxic activity against MDA-MB-468 and PC-12 cells with IC_50_ values of 0.6±0.04 µM and 0.43±0.06 µM, respectively. On treatment with the 5c derivative, all cell lines showed an increased rate of cell death at a lower concentration among other derivatives (Figure 2 and ​Table 2).

***TUNEL test***

**Figure 3:**
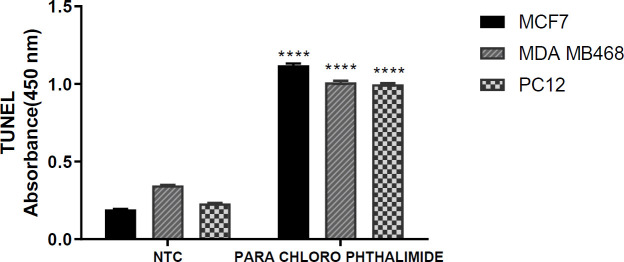
TUNEL assay for cell apoptosis comparison in three cancer cell lines (MCF-7, MDA-MB468, and PC-12). The results were expressed as Mean±SEM of three independent experiments (n=3) in comparison with the positive control group (3 cell lines treated with nuclease solution) and the negative control group (3 cell lines treated with DMSO). (* Indicates *P*-value<0.05, ** indicates *P*-value <0.01, *** indicates *P*-value<0.001 and **** indicates *P-* value < 0/0001)

TUNEL test that was utilized for detection of the apoptotic cells, showed an increase in these cells in all cancer cell lines compared with the control group after exposure to the** 5c** derivative. Compared with the negative control group, this compound significantly increased DNA damage in MCF-7 cells, which indicates the induction of apoptosis by this compound (Figure 3).

***Caspase-3 activity analysis***

**Figure 4:**
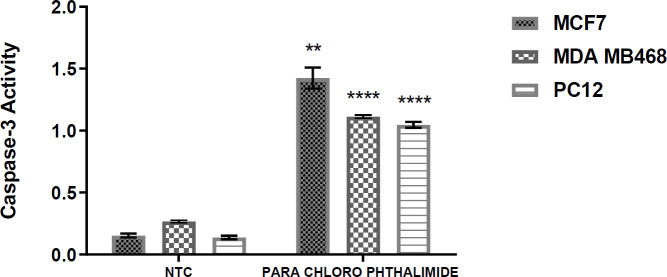
Caspase-3 activity in cancer cell lines treated with **5c** compound. MCF7, MDA-MB-468, and PC12 cell lines were treated for 24 hr with a **5c** compound. Enzymatic activities of caspase-3 activities in treated and untreated cells were then determined by the spectrophotometer. The results were expressed as Mean±SEM of three independent experiments (n=3) in comparison with the positive control group (3 cell lines treated with nuclease solution) and the negative control group (3 cell lines treated with DMSO). (* Indicates *P*-value <0.05, ** indicates *P*-value<0.01, *** indicates *P*-value<0.001 and **** indicates *P -*value<0/0001)

Caspases-3 activity was analyzed on cancer cells following treatment with 5c derivative. This work was carried out to confirm that induced cytotoxicity was due to apoptosis. Cells were treated with IC_50_ of **5c** derivative and analyzed to detect the activity of caspase-3 (Figure 4). As expected, all cell lines treated with the **5c** derivative showed increased caspase-3 activity. Compared with the negative control group, this compound significantly increased caspases-3 activity in MCF-7 and PC-12 cells, which indicates induction of apoptosis by this compound.

***Changes of genes expression***

**Figure 5:**
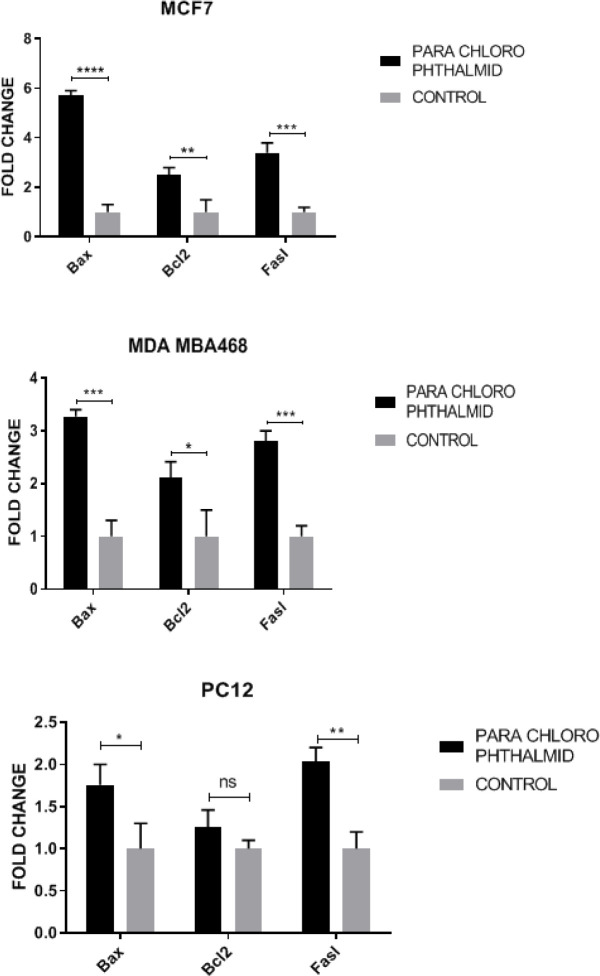
Comparison of the fold changes in RNA expression levels of BAX, BCL-2 and FAS genes in A) MCF7 (*P-*value bax =0.000023) B) MDA-MB468 (*P-*value bax= 0.000274 and *P-*value fasl= 0.000385) and C) PC12 (*P-* valu bax= 0.0266 and* P*-value fasl= 0.0021) cell lines after treatment with IC_50 _concentration of **5c **compound for 24 hr

Expression of Bax, Bcl-2, and FasL genes and the GAPDH gene as internal control were investigated in 24 hr treatment with the **5c** derivative. Expression of Bax, Bcl-2, and FasL genes showed an increase in all cell lines compared with the control group. Compared with the negative control group, this compound significantly increased expression of the Bax gene in the MCF-7 cell line. Transcript expression of both Bax and FasL genes increased significantly and closely (Figure 5). 

**Table 1:** Physicochemical properties of compounds 5a-5k

R	Compound	Chemical Formula	MW (g/mol)	mp (°C)	Yield (%)
**4-F**	**3a**	C_9_H_7_FN_2_S	194	95	83
**2-Cl**	**3b**	C_9_H_7_ClN_2_S	210	124	86
**4-Cl**	**3c**	C_9_H_7_ClN_2_S	210	150	89
**2-OCH**_3_	**3d**	C_10_H_10_N_2_OS	206	173	81
**3-OCH**_3_	**3e**	C_10_H_10_N_2_OS	206	105	83
**4-OCH**_3_	**3f**	C_10_H_10_N_2_OS	206	188	90
**2-OH**	**3g**	C_9_H_8_N_2_OS	192	96	61
**3-OH**	**3h**	C_9_H_8_N_2_OS	192	155	63
**4-OH**	**3i**	C_9_H_8_N_2_OS	192	160	68
**4-NO**_2_	**3j**	C_9_H_7_N_3_O_2_S	221	260	87
**4-Br**	**3k**	C_9_H_7_BrN_2_S	255	157	89
**4-F**	**5a(K)**	C_19_H_12_FN_3_O_3_S	381	94-99	75
**2-Cl**	**5b(B)**	C_19_H_12_ClN_3_O_3_S	397	95	67
**4-Cl**	**5c(A)**	C_19_H_12_ClN_3_O_3_S	397	67-69	83
**2-OCH**_3_	**5d(H)**	C_20_H_15_N_3_O_4_S	393		76
**3-OCH**_3_	**5e(D)**	C_20_H_15_N_3_O_4_S	393	195	65
**4-OCH**_3_	**5f(J)**	C_20_H_15_N_3_O_4_S	393	140	73
**2-OH**	**5g(E)**	C_19_H_13_N_3_O_4_S	379	73-75	68
**3-OH**	**5h(F)**	C_19_H_13_N_3_O_4_S	379	108-110	81
**4-OH**	**5i(C)**	C_19_H_13_N_3_O_4_S	379	112-115	70
**4-NO**_2_	**5j(I)**	C_19_H_12_N_4_O_5_S	408	70	84
**4-Br**	**5k(G)**	C_19_H_12_BrN_3_O_3_S	442	102-104	79

**Table 2:** IC_50_ (μM) values for the *in vitro* cytotoxic activity of 11 phthalimide derivatives on three human cancer cell lines

R	Compound	MDA-MB468	PC12	MCF7
**4-F**	**5a(K)**	4±0.9	1.7±0.74	ND*
**2-Cl**	**5b(B)**	0.67±0.31	2.7±0.09	0.2±0.01
**4-Cl**	**5c(A)**	0.63±0.02	0.7±0.04	0.4±0.03
**2-OCH**_3_	**5d(H)**	0.87±0.17	1.2±0.06	4.7±0.37
**3-OCH**_3_	**5e(D)**	2.6±0.12	0.58±0.02	4.76±0.43
**4-OCH**_3_	**5f(J)**	1.3±0.09	0.8±0.05	0.5±0.07
**2-OH**	**5g(E)**	1.03±0.09	0.43±0.06	0.2±0.05
**3-OH**	**5h(F)**	0.7±0.04	0.65±0.06	1.18±0.04
**4-OH**	**5i(C)**	0.8±0.05	1.6±0.4	0.8±0.06
**4-NO**_2_	**5j(I)**	1.2±0.02	0.7±0.08	1.6±0.07
**4-Br**	**5k(G)**	0.6±0.04	0.8±0.05	1.7±0.09
**Doxorubicin**	-	0.38±0.07	2.6±0.13	2.63±0.4

*ND: not done

## Discussion

***Structure activity relationship (SAR)***

A novel series of phthalimide-based compounds incorporated into the 1,3-thiazole nucleus was designed (Figure 1) and subsequently, the anticancer properties and capabilities were investigated *in vitro*. Both of the electron-donating (OH, -OCH_3_) and electron-withdrawing (F, Cl, Br, NO_2_) substituents were applied on the phenyl residue to control the various electronic as well as steric effects. Fortunately, promising results were deduced against tested cell lines while comparison was carried out with doxorubicin as a standard anticancer drug (Table 2). Three cancerous cell lines that were utilized were MDA-MB468, PC12, and MCF-7. Although, interesting results were obtained from the tested compounds against MDA-MB468 cells, but tested derivatives displayed inferior potency compared with doxorubicin (IC_50_ = 0.38 ± 0.07 µM). The most potent compounds demonstrated a cytotoxic potency of less than 1 µM towards MDA-MB468 cells. Halogenated derivatives with chlorine and bromine moieties (**5b, 5c, 5k**) and also two out of three hydroxylated derivatives (**5h, 5i**) exhibited potency in the nanomolar range (IC_50_ > 1 µM). Both electron-donating and electron-withdrawing effects were beneficial parameters for cytotoxic enhancement against MDA-MB468 cells. 

PC12 and MCF-7 cells were so sensitive to tested derivatives. Amazing results were observed due to the cytotoxic evaluation of these compounds against the mentioned cell lines. All of the assessed derivatives showed more cytotoxic effects against PC12 cells than doxorubicin (IC_50_ = 2.6 ± 0.13 µM) except for compound **5b** (IC_50_ = 2.7 ± 0.09 µM). Compound **5g** with *ortho* hydroxyl moiety was the most potent compound in these series against PC12 cells. Electron releasing property of the hydroxyl group and also the capability for hydrogen bonding interaction are likely responsible factors for increasing the potency. 

Synthesized derivatives **5a-5k **demonstrated outstanding cytotoxic effects towards MCF-7 cells. Most of the evaluated compounds caused stronger cytotoxic results against this cell line compared with doxorubicin except for *para* fluorinated and *ortho/meta* methoxylated derivatives. The same results were obtained for the other cell lines, both of the electron-donating and electron-withdrawing groups influenced the cytotoxic activity of this chemical structure towards positive outcomes for MCF-7 cells. In fact, chlorinated (compound **5b**, 2-Cl, IC_50_ = 0.2 ± 0.01 µM) and hydroxylated (compound **5g**, 2-OH, IC_50_ = 0.2 ± 0.05 µM) derivatives were the most active compounds in these series. 

***Biological evaluation***

Cell viability and growth inhibition of 11 phthalimide derivatives were studied on three cancer cell lines including MCF-7, MDA-MB-468, and PC-12. On the basis of the above results, cell viability assessment was performed by measuring the levels of live cells after incubation with different doses of phthalimide derivatives for 48 hr, using the MTT colorimetric assay. The cell viability results are presented in Figure 2 and Table 2. Compound 5c gave rise to a significant change in cell viability in all cancer cell lines at IC_50_ concentration, compared with the standard drug doxorubicin and other compounds. Compound **5c**, bearing a 4-Cl moiety, could be responsible for a significant decrease in cell viability compared with other compounds. The growth inhibitory property (IC_50_) for compound **5c** was 0.4±0.03 µM in MCF-7, 0.63±0.02 µM in MDA-BM-468 and 0.7±0.04 µM in PC-12.

In the present study, we aimed at investigating whether the effect of compound **5c **occurred via the apoptotic pathway and, if so, which apoptotic pathway was related to this process. Apoptosis is one of the major mechanisms of cell death in response to cancer therapies. Signaling for apoptosis occurs by multiple independent pathways that are initiated by different extracellular and intracellular factors. The intrinsic cell death pathway is also known as the mitochondrial apoptotic pathway (39, 40). 

In this study, the effects of compound **5c** on apoptosis were investigated via assessing Bcl-2, Bax and Fas-L, and caspase-3 levels. Tumor cell apoptosis is regulated by interaction of numerous tumor suppressor genes and oncogenes, such as Bcl-2 and Bax. The oncogene Bcl-2 induces cell survival, although the tumor suppressor gene Bax induces cell death. Both represent important factors in the control of the apoptotic pathway. Both can form homodimers and heterodimers, and the balance between the respective dimers (Bcl-2/Bcl-2; Bcl-2/Bax; Bax/Bax) determines the extent to which apoptosis is induced or suppressed. It has been proposed that Bax homodimers promote apoptosis and that Bax-mediated cell death is counteracted by Bcl-2/ Bax heterodimerization (4). Thus, the Bcl-2/Bax ratio represents one cell-autonomous mechanism that determines the cell’s fate. Another mechanism by which cells can be removed is the Fas pathway. The Fas system belongs to the tumor necrosis factor (TNF) family, which consists of numerous ligands that bind to their homologous receptors. The apoptosis-inducing receptor Fas, when activated by its ligand, Fas ligand (Fas-L), trans-duces the death signal through a highly conserved cytoplasmic ‘death domain’. Fas is a 45 kDa type I membrane protein belonging to the TNF-receptor family (11, 12). Expression of Fas and Fas-L is not only restricted to lymphoid tissue or immune-privileged organs but is also found in various epithelial tissues (13). In tumor cells, the expression of Fas-L is involved in the tumor immune escape mechanism (14, 15). However, in many tumors a co-expression of Fas receptor and FasL can be found, indicating that tumor cells are also suggestible to Fas-induced apoptosis (41-49). Caspase-3 induces the typical apoptosis features, including DNA fragmentation and cell death in several tissues. The mitochondrial pathway is relatively influenced by bcl family members bound to the mitochondrial membrane, including bax and bcl-2, which are, respectively, pro- or antiapoptotic regulatory proteins (50).

After the cells were treated with compound **5c**, Bcl-2, Bax, and FasL levels in all cancer cell lines were greater than those of the control group (Figure 5). Caspase-3 levels are shown in Figure 4. The compound **5c** caused an increase in the caspase-3 activity as compared with the control group (in all of the three cells). In this study, we investigated the effects of compound **5c** on the intrinsic apoptotic markers (Bcl-2, Bax, and caspase-3 activation) and Fas-L expression (as marker for the point of convergence of the extrinsic apoptotic pathways) in MCF-7, MDA-MB-468, and PC-12 cell lines. Our data showed that treatment with compound **5c** directly up-regulated Bcl-2, Bax, and Fas-L expression and induced caspase-3-dependent apoptosis in MCF-7, MDA-MB-468, and PC-12 cell lines. Our findings indicated that compound **5c** induced apoptosis in human MCF-7, MDA-MB-468, and PC-12 cancer cell lines through the intrinsic and extrinsic apoptotic pathways.

## Conclusion

In summary, *para* chloro derivative of synthesized compounds **(5c)** caused an increase in cytotoxicity, TUNEL assay, caspase-3 activity. In addition, up-regulation of Bax, Bcl-2, and Fas-L mRNA levels in MDA-MB-468, PC-12, and MCF-7 was observed in cancer cell lines. Collectively, these findings suggest that *para* chloro derivative** (5c) **induces an apoptotic effect via intrinsic and extrinsic pathways in these cancer cells as a potent anticancer agent.
